# Evaluation of quality of life of gastrointestinal cancer patients presenting to a tertiary care hospital in Pakistan

**DOI:** 10.3332/ecancer.2023.1527

**Published:** 2023-04-03

**Authors:** Muhammad Tayyab H Siddiqui, Muhammad Rizwan Khan, Adnan Jawaid, Fatima Shaukat, Nida Zahid

**Affiliations:** 1 Department of Surgery, Patel Hospital, Karachi 75300, Pakistan; 2 Department of Surgery, Aga Khan University Hospital, Karachi 74000, Pakistan; 3 Department of Radiation Oncology, Cyber-knife and Tomotherapy Centre, Jinnah Post-Graduate Medical Center, Karachi 75510, Pakistan

**Keywords:** quality of life, gastrointestinal cancer, EORTC QLQ-C30

## Abstract

**Introduction:**

Quality of life (QOL) appraisal is a meaningful method of outcomes assessment in patients with gastrointestinal (GI) cancer. The aim of our study was to evaluate QOL of patients suffering from GI cancer, who underwent treatment at Aga Khan University Hospital (AKUH), Karachi, Pakistan.

**Methods:**

It was a cross-sectional study. A total of 158 adults from December 2020 to May 2021 were included in the study. The EORTC QLQ-C30, validated in Urdu (Pakistan) version, was used to assess the QOL of the participants. Mean QOL scores were calculated and compared with threshold of clinical importance (TCI). Multivariate analysis was done to analyse the correlation between independent factors and QOL scores. A p value of <0.05 was considered as significant.

**Results:**

Mean age of the study participants was 54.5 ± 13 years. Majority were male, married and living in combined family system. Most common GI cancer was colorectal (61%) followed by stomach (33.5%); and the most frequent stage at presentation was stage III (40%). Global QOL score was found to be 65.48 ± 1.78. Among functioning scales, role functioning, social functioning, emotional functioning and cognitive functioning were found to be above TCI, whereas physical functioning was found to be below TCI. Among symptom scores, fatigue, pain, dyspnoea, insomnia, appetite loss, constipation and diarrhoea were found to be below TCI, whereas nausea/vomiting and financial impact were found to be above TCI. Multivariate analysis revealed that history of surgery had a positive association (*p* < 0.001), while being on treatment (*p* = 0.001) and having a stoma (*p* = 0.038) had a negative impact on global QOL.

**Conclusion:**

This is the first study to evaluate the QOL scores in GI cancer patients in Pakistan. There is a need to identify the reasons for low physical functioning score and explore means to mitigate symptoms scores above TCI in our population.

## Introduction

Cancer is a leading cause of death in many countries and expected to be the most common reason of decreasing life expectancy worldwide over the course of this century [1]. Likewise, the rate of cancer is escalating in Pakistan, with an expected rise in the incident rate of 150,000 cases each year [2]. Gastrointestinal (GI) cancers allude to the malignancies emerging in the GI tract and accessory organs involved in digestion [3].

GI cancer and its treatment have a noteworthy impact on wellbeing related to the QOL [4]. Chemotherapy instigated nausea and emesis, surgical procedure related altered bowels habits and need of dietary modifications, social and emotional trauma because of stoma bag and skin irritation post radiation are few of many sufferings faced by GI cancer patients. Calculating morbidity, mortality, recurrence rate, overall and disease free survival are the classic methods to assess the success of a treatment plan.

Over the years, quality of life (QOL) appraisal has assumed a significant importance in the assessment of chronic disease, especially the cancer clinical preliminaries [5]. As of late, different tools have been created to quantify significant result factors for evaluating the QOL in cancer patients, especially self-managed questionnaires [6]. The European Organization of Research and Treatment of Cancer (EORTC) has built up a centre QOL questionnaire QLQ-C30, which is the most generally utilised cancer questionnaire [7]. EORTC-QLQ-C30 is deciphered and approved in excess of 60 dialects including Urdu [7]. Numerous clinical studies performed to assess the QOL in patients suffering from cancer have been conducted which demonstrate that different elements like age, co-morbid conditions and education have an integral impact on QOL of patients diagnosed with cancer [8].

In Pakistan, only few studies to assess QOL have been conducted in patients with breast cancer and patients undergoing chemotherapy [9–11]. However, no study has so far been reported to examine the overall QOL in GI cancer patients and its related factors. The objectives of our study were to evaluate the QOL of patients suffering from GI cancer using EORTC QLQ-C30 (Urdu) and to identify factors associated with QOL of GI cancer patients. It is a basic prerequisite to assess QOL of GI cancer sufferers, to make fitting strides towards their ideal care.

## Methodology

An analytical cross-sectional study was designed and Institutional Ethical Review Committee (ERC) approval was taken before collecting the data. Patients aged 18 years or above, who were receiving or previously received any form of treatment for GI tumour at AKUH and willing to participate in the study were approached in surgery and oncology clinics. Patients with prior history of malignancy other than GI cancer, patients with psychiatric disorders or those who are unable to understand English or Urdu were excluded. Written and verbal consent was taken from eligible patients who were willing to participate. Each participant was provided with two self-filled questionnaires. Either patient or caregiver filled the questionnaire, help was provided by one of the trained team member whenever needed. Socio-demographic and factors associated with QOL like age, gender, education, marital status, disease status and treatment received were collected in a self-designed questionnaire. For QOL assessment, EORTC QLQ-C30 Urdu (Pakistan) version was used.

The EORTC QLQ-C30, validated in Urdu (Pakistan) version is a core cancer specific questionnaire. It consist of 30 items measuring functioning scales (physical, social, role, cognitive and emotional), disease and treatment related symptoms scales (fatigue, nausea/vomiting, pain, dyspnoea, sleep disturbance, appetite loss, constipation, diarrhoea and financial impact) and global QOL. The scores marked by each participant for every scale were converted to 0–100 scales according to EORTC scoring manual [12]. For functioning scale and global QOL, higher score indicates better QOL whereas for symptom scales higher scores indicate higher symptom burden, i.e. worse QOL. All measured scores for functioning scales and symptom scales were compared with threshold of clinical importance calculated by EORTC [13].

The sample size was calculated based on previously reported estimates for QOL among patients with GI cancers. It has been calculated using one population mean formula, based on a SD range of 7.5%–25.9%, 5% level of significance with precision of 5 and by adjusting the sample size for 10% non-response rate. The minimum sample size is estimated to be 113 [14–17].

Data were analysed on STATA version 15. The quantitative variables are reported as mean± SD/ median (IQR). The qualitative variables are reported as frequency and percentages. Mean scores calculated for different variables of QOL . To analyse the correlations between the independent factors and the QOL scales, general linear model (GLM) multivariate analysis of variance (MANOVA) is performed. Univariate and multivariate Wilks is reported to test the impact of each variable included in the model. To assess the relationship of global QOL with the independent factors, linear regression modelling is performed and unadjusted and adjusted beta coefficient is reported with their 95% CI. A *p* value of <0.05 is considered as significant throughout the study.

## Results

Overall, 158 GI cancer patients participated in this study. Table 1 represents the demographic profile of participants. Mean age of the study participants was 54.5 ± 13 years. Majority were male (73%), married (86%), living in combined family system (55%). Only 65 (41%) of the study participants were employed. Literacy status revealed that 77 (48.7%) participants were graduates, while 30 (19%) were either illiterate or just able to write their names. Urdu (29.7%) was the most commonly speaking language in study participants followed by Sindhi (25.3%).

Table 2 summarises clinical characteristics and treatment received by study participants. Most common cancer included colon 76 (48%) followed by stomach 53 (33.5%). Stage III was the most frequent stage at presentation (40%). Surgical intervention was performed in 127 (80.4%) patients, out of which 39 (24.7%) had a stoma. Majority of the patients (61.4%) were on treatment at the time of participation in the study. The most common comorbid found in our participants was hypertension 60 (38%) followed by diabetes mellitus 44 (27.8%).

The overall global QOL score was 65.48 ± 1.78. Individual domain scores are presented in Table 3 with TCI scores for comparison. It was observed that among functioning scales, role functioning, social functioning, emotional functioning and cognitive functioning were found to be above TCI whereas physical functioning was found to be below TCI. Among symptom scores; fatigue, pain, dyspnoea, sleep disturbance, loss of appetite, constipation, diarrhoea were found to be below TCI, whereas nausea/vomiting and financial impact were found to be above TCI.

Linear regression model was used to assess the association between factors affecting global QOL. Table 4 shows the univariate and multivariate analysis. Multivariate analysis showed that patients who had surgery (*p* < 0.001) had a significant better global QOL whereas those patients who had a stoma (*p* = 0.001) or who are currently on treatment (*p* = 0.038) had a significantly worse global QOL.

GLM-MANOVA was used to determine the association between the independent variables and the dependent variables (15 scales of QLQ–C30). On the one-factor model, 12 variables were associated with the overall outcome of QLQ–C30 with *p* < 0.20. These 12 variables were entered into the multifactor model analysis. Variables significantly associated on the multifactor model were functional capacity, past surgical history, grade of tumour and recurrence of tumour (all *p* < 0.05). Results of the GLM-MANOVA are shown in Table 5.

## Discussion

We analysed the QOL of GI cancer patients using Urdu version of EORTC QLQ C30 tool, one of the most widely used patient reported outcome tool to assess health related QOL [18]. The mean global QOL score was 65.48 ± 1.78. Our Global QOL score was found to be comparable to international studies on GI cancer patients ranging from (55–76.8) [19–22]. When we compared our global QOL score with other studies on Pakistani population, we observe a better score in our GI cancer patients as compared to patients with blood cancer (25.95), breast cancer (48.33) and with different combination of cancer on chemotherapy (57.37) [9–11]. These differences can be explained by complete different spectrum of symptomatology, treatment options especially chemotherapeutic drugs and problems associated with those treatment modalities.

Among the five functioning scales, physical functioning was the most commonly affected area in our patients with a mean score of 75.63 ± 1.83 which is comparable to the scores from the international data on GI cancers which ranges from 60 to 92 [19–23]. This high variability is likely due to the variable stage of disease and the mode of treatment. Lowest reported scoring scale among functioning scale is variable in literature: social functioning in Japan [21], role functioning in Sweden and China [19, 22] and emotional scale in Columbia and the USA [20, 23]. Due to inadequate infrastructure and limited expertise in minimally invasive approach to deal with cancer patients, open surgery is the main option for GI cancer surgery. This could be the reason of low physical functioning score, as majority of our patients underwent an open surgical procedure.

Among the nine symptom scales, financial impact was found to be the worst (42.94) followed by fatigue (30.21) and appetite loss (26.11). Sánchez *et al* [20] reported fatigue (44.5) as the worst parameter in gastric cancer and financial impact (36.3) as the worst parameter in colorectal cancer patients. As per Matsushita *et al* [21], sleep disturbance was with the worst score in symptoms scale. Fatigue was with the highest score in symptom scale as per Nordin *et al* [22]. The highest score with financial impact in our part was expected as majority of patients pay from their pocket and no healthcare uniformity exist for citizen from government. We are a private tertiary care hospital with state-of-the-art methods of treating cancer patients, so there is a selection bias and it may not be representative of all population. This is an area of very high importance and least discussed and needs further work. Many reforms are being introduced like heath cards which should improve this figure in near future.

Interpretation of the mean scores into meaningful clinical importance was a challenge. As majority of the work done was based on expert opinion, EORTC group systematically developed threshold for clinical importance (TCIs) after including the opinion of both patient and health professionals [13]. These thresholds have further increased the practicality of the questionnaire in real-life clinical scenarios. Table 3 describes the comparison of five functioning scales and nine symptom scales with TCI. Among functioning scales: Physical functioning was found to be below TCI while rest of the functioning scales were above TCI. Among symptom scales: financial impact and nausea/vomiting were two scales that were above TCI. We can appreciate that fatigue and appetite loss were ranked second and third in terms of worse symptom scale scores but their values were below TCI, and nausea/vomit ranked seventh but the score was found to be above TCI. So it is important to understand the value of the score and target to compare to in order to identify the areas where urgent focus is required to improve QOL in GI cancer patients. The reason for nausea/vomiting to be above TCI in GI cancer patients is not only due to the disease but also treatment modalities. Chemotherapy, radiation and surgery all affect GI motility.

Multiple factors affect the QOL in cancer patients including age, gender, marital status, employment status, level of education, nutritional status, presence of comorbidities, tumour location and stage of cancer [10, 11, 20, 23, 24]. In our study, patients who underwent surgery had a significantly better global QOL when compared with those who didn’t undergo any procedure. These findings are consistent with Calderón *et al* [26] and Amemiya *et al* [25]. who reported that patients with colorectal cancer and stomach cancer exhibited a better QOL post-surgery. This finding could be due to the fact that majority of our patients had colorectal cancers. Similar to existing literature, our study also showed that patients who are currently on treatment, i.e., adjuvant chemotherapy, had a poor global QOL. Chemotherapy, an essential component of cancer management comes with significant distress to patient at various stages of treatment plan [11, 27]. Multiple studies have demonstrated that presence of stoma is associated with poor QOL [28, 29]. Likewise our study shows that patients who had a stoma in their treatment plan had a significantly poor global QOL score. This finding was not surprising as having a stoma is a major concern in our population due to religious beliefs and adversely affects the QOL [30].

Functional capacity, documented as the Eastern Cooperative Oncology Group (ECOG) scale in our study, has a significant impact on QOL parameter. As per Schag *et al* [31], performance status is the best indicator of QOL. Likewise, in our analysis of all 15 scales combined, functional status of an individual was found to be a significant factor. History of previous surgery and higher grade of tumour were also found to be contributing to affect the QOL in our study but no such correlation is reported in available literature. Recurrence of tumour be it any site worsens the QOL of patients, studies have reported that even fear of recurrence has a significant impact on QOL of cancer patients [32, 33]. Multifactor model identified that those patients who had a recurrence has a significantly worse QOL and our findings are consistent with available literature [34, 35].

## Conclusion

GI cancer symptoms and treatment plans vary as per the site of tumour, extending from oesophagus to the anal canal. Although EORTC have developed site-specific tools to assess QOL as per the site of tumour, we used EORTC QLQ C30 as its validated Urdu version was already available and few site-specific tools were in the process of translation into our native language. This is the first study from our region with the aim to develop baseline scores and factors severely affecting GI cancer patients in our community. A potential way forward would be to transform techniques to improve factors already identified, to plan multicentre studies representative of the whole population specially public sector and to perform site-specific studies using site-specific tools to establish means to improve QOL of our cancer patients in greater depth.

## Conflicts of interest

No conflict of interest of any author.

## Figures and Tables

**Table 1.** Demographic profile of GI cancer patients.

Characteristics		Frequency	Percent
	Total	158	
Age	Mean ± SD	54.54 ± 13.23	
Gender	Male	116	73.4
	Female	42	26.6
Mother language	Urdu	47	29.7
	Sindhi	40	25.3
	Punjabi	14	8.9
	Balochi	14	8.9
	Pathan	10	6.3
	Others	33	20.9
Educational status	Illiterate	16	10.1
	Can write name	14	8.9
	Primary	16	10.1
	Matriculation	9	5.7
	Intermediate	26	16.5
	Graduate	77	48.7
Marital status	Unmarried	12	7.6
	Married	136	86.1
	Divorced/widowed	10	6.4
Family type	Nuclear	69	43.7
	Combined	87	55.1
No of family members	Median (IQR)	7(4)	
Employment status	Employed	65	41.1
	Employed but unable to work due to disease	18	11.4
	Retired	42	26.6
	Housewife	28	17.7
Monthly family income	Median (IQR) in Pak Rupees	70,000 (122,500)	
Functional class (**ECOG**)	Fully active	106	67
	Active with some difficulty	22	14
	Able to self-care	12	7.6
	Needs assistance in self-care	11	7
	Totally dependent	1	0.6
	Not reported	6	3.8
History of past surgery		69	44

**Table 2.** Clinical characteristics and treatment received.

Characteristics		Frequency	%
Site of tumour	Colon	76	48.1
	Stomach	53	33.5
	Rectum	21	13.3
	Liver	6	3.8
	Oesophagus	1	0.6
	Pancreas	1	0.6
Stage of disease	I	10	6.3
	II	42	26.6
	III	63	39.9
	IV	23	14.6
Grade of tumour	0	19	12.0
	Well differentiated	18	11.4
	Moderately Differentiated	76	48.1
	Poorly differentiated	45	28.5
On treatment		97	61.4
Neoadjuvant treatment		45	28.5
Surgical intervention		127	80.4
Adjuvant treatment		104	65.8
Stoma bag created		39	24.7
Type of stoma	Ileostomy	23	14.6
	Colostomy	17	10.8
Nature of stoma	Temporary	25	15.8
	Permanent	16	10.1
Recurrence		16	10.1
Comorbids	Hypertension	60	38
	Diabetes mellitus	44	27.8
	Ischaemic heart disease	17	10.8
	Chronic kidney disease	5	3.2
	Hepatitis	14	8.9
Smoking status	Never	108	68.4
	Ex-smoker	42	26.6
	Active smoker	7	4.4
Other addictions	Alcohol	6	3.8
	Betel nut	5	3.2
	Tobacco	7	4.4
	Naswar	7	4.4
	Others	3	1.9
BMI category	Underweight	11	7
	Normal	66	41.8
	Over weight	42	26.6
	Obese	28	17.7
BMI	Mean ± SD	25.66 ± 5.71	

**Table 3.** QOL score and comparison with threshold of clinical significance (TCI).

Domains		Mean score	SD	TCI
**Global**		65.48	1.78	
**Functioning scale**	Physical	75.64	1.83	83
	Social	80.98	1.92	58
	Role	83.76	1.98	58
	Cognitive	88.35	1.26	75
	Emotional	82.42	1.65	71
**Symptom scale**	Fatigue	30.21	1.97	39
	Nausea/vomiting	13.26	1.83	8
	Pain	18.69	1.94	25
	Dyspnoea	12.95	1.84	17
	Sleep disturbance	23.14	2.2	50
	Appetite loss	26.11	2.59	50
	Constipation	21.86	2.53	50
	Diarrhoea	12.52	2.02	17
	Financial impact	42.94	3.09	17

**Table 4.** Univariate and multivariate analysis for assessing factors associated with Global QOL.

Variables		Coef.	Std. Err.	95% CI
Univariate				
Family no.*		0.45	0.26	−0.06 to 0.96
Employment status* Employed (ref)	Employed but unable to work	−12.72	6.02	−24.62 to −0.81
	Retired	3.76	4.41	−4.95 to 12.48
	Housewife	−1.34	5.06	−11.34 to 8.66
Surgery*		16.33	4.28	7.86 to 24.79
Adjuvant treatment*		5.25	4.17	−3 to 13.5
On treatment*		−12.45	3.66	−19.7 to −5.2
Neo-adjuvant treatment*		−6.29	3.94	−14.09 to 1.5
Grade of tumour* Well-differentiated (ref)	Moderately differentiated	11.21	5.67	0.00029 to 22.43
	Poorly differentiated	−0.93	6.02	−12.85 to 10.98
Stage of tumour* Stage I (ref)	Stage II	10.63	7.35	−3.91 to 25.17
	Stage III	2.5	7.11	−11.56 to 16.56
	Stage IV	−11.99	7.89	−27.61 to 3.62
Stoma*		−5.82	4.38	−14.52 to 2.88
Multivariate analysis				
Surgery**		2.12	0.42	1.28 to 2.97
On treatment**		−0.85	0.24	−1.33 to −0.37
Stoma**		−0.51	0.24	−1 to −0.02

* *p* value < 0.2 on univariate analysis

** *p* value < 0.05 on multivariate analysis

NS, Not significant

**Table 5.** Association of clinico-demographic variables with QLQ-C30 using GLM-MANOVA.

Variables	One factor model Wilk λ (*p* value)	Multifactor model Wilk λ (*p* value)
Age	0.76 (<0.001) *	NS
Marital status	0.67 (0.15) *	NS
No of household family members	0.86 (0.13) *	NS
BMI	0.85 (0.16) *	NS
Functional capacity	0.32 (<0.001) *	0.29 (<0.001) **
Chronic kidney disease	0.86 (<0.19) *	NS
Liver problem	0.87 (0.19) *	NS
Past surgical history	0.84 (0.05) *	0.76 (<0.02) **
Type of tumour	0.52 (0.09) *	NS
Grade of tumour	0.60 (0.007) *	0.55 (0.04)**
On treatment	0.78 (0.005) *	NS
Recurrence	0.77 (<0.01) *	0.76 (0.01)**

* *p* value < 0.2 by one factor model of GLM-MANOVA

** *p* value < 0.05 by multifactor model of GLM-MANOVA

GLM-MANOVA, General linear model multivariate analysis of variance; NS, Not significant
